# Corticosteroid suppression of lipoxin A_4 _and leukotriene B_4_from alveolar macrophages in severe asthma

**DOI:** 10.1186/1465-9921-11-71

**Published:** 2010-06-07

**Authors:** Pankaj K Bhavsar, Bruce D Levy, Mark J Hew, Michael A Pfeffer, Shamsah Kazani, Elliot Israel, Kian Fan Chung

**Affiliations:** 1 Experimental Studies, Airways Disease Section, National Heart & Lung Institute, Imperial College London & Royal Brompton NHS Trust, London, UK; 2 Pulmonary and Critical Medicine, Brigham and Women's Hospital and Harvard Medical School, Boston, MA, USA

## Abstract

**Background:**

An imbalance in the generation of pro-inflammatory leukotrienes, and counter-regulatory lipoxins is present in severe asthma. We measured leukotriene B_4 _(LTB_4_), and lipoxin A_4 _(LXA_4_) production by alveolar macrophages (AMs) and studied the impact of corticosteroids.

**Methods:**

AMs obtained by fiberoptic bronchoscopy from 14 non-asthmatics, 12 non-severe and 11 severe asthmatics were stimulated with lipopolysaccharide (LPS,10 μg/ml) with or without dexamethasone (10^-6^M). LTB_4 _and LXA_4 _were measured by enzyme immunoassay.

**Results:**

LXA_4 _biosynthesis was decreased from severe asthma AMs compared to non-severe (p < 0.05) and normal subjects (p < 0.001). LXA_4 _induced by LPS was highest in normal subjects and lowest in severe asthmatics (p < 0.01). Basal levels of LTB_4 _were decreased in severe asthmatics compared to normal subjects (p < 0.05), but not to non-severe asthma. LPS-induced LTB_4 _was increased in severe asthma compared to non-severe asthma (p < 0.05). Dexamethasone inhibited LPS-induced LTB_4 _and LXA_4_, with lesser suppression of LTB_4 _in severe asthma patients (p < 0.05). There was a significant correlation between LPS-induced LXA_4 _and FEV_1 _(% predicted) (r_s _= 0.60; p < 0.01).

**Conclusions:**

Decreased LXA_4 _and increased LTB_4 _generation plus impaired corticosteroid sensitivity of LPS-induced LTB_4 _but not of LXA_4 _support a role for AMs in establishing a pro-inflammatory balance in severe asthma.

## Introduction

Patients with asthma are usually well-controlled with inhaled corticosteroids (CS) and long-acting β_2_-agonists, but a minority of patients described as severe asthma continues to experience uncontrolled asthma in spite of these treatments. Patients with severe asthma suffer greater morbidity, face a higher risk of asthma death, and consume a greater proportion of health resources than other non-severe asthma patients [1,2]. One feature of severe asthma is the presence of airway inflammation despite corticosteroid therapy, often characterised by the persistence of eosinophilic inflammation and the presence of neutrophils[3,4]. Persistent symptoms with frequent exacerbations of asthma despite corticosteroid therapy also indicate the possibility that CS may not be as effective in patients with severe asthma. The presence of reduced CS sensitivity in severe asthma is supported by the finding that release of cytokines from peripheral blood mononuclear cells and alveolar macrophages is less suppressible by dexamethasone than those from non-severe asthma patients[5,6].

Lipid mediators of the 5-lipoxygenase pathway such as cysteinyl-leukotrienes are implicated as mediators of airway bronchoconstriction and eosinophilic inflammation in asthma; another product, leukotriene B_4 _(LTB_4_), has also been implicated, particularly in view of its chemoattractant and activating properties for neutrophils [7]. Similar to LTs, lipoxins (LXs) are products of arachidonic acid metabolism, yet LXs are generated via interactions between 5- and 15-lipoxygenases or 5- and 12-lipoxygenases to form structurally distinct compounds that promote the resolution of inflammation. Thus, LXs are counter-regulatory to the cysteinyl-leukotrienes and LTB_4 _[8]. The possibility that dysregulation of the balance among these arachidonic acid products might contribute to the persistent inflammation in severe asthma has been supported by the demonstration of an increased generation of cysteinyl-leukotrienes with impaired biosynthesis of lipoxin A_4 _(LXA_4_) from whole blood of patients with severe asthma compared to non-severe asthma patients[9]. In addition, LXA_4 _levels in bronchoalveolar lavage fluid of patients with severe asthma from the NHLBI Severe Asthma Research Program were decreased when compared to non-severe asthma patients [10].

We determined whether an imbalance in pro-inflammatory LTB_4 _and anti-inflammatory LXA_4 _in the lungs of patients with severe asthma could be reflected in the formation of these products from alveolar macrophages (AMs). We also determined whether there would also be a differential suppressibility of these mediators that reflect different effects in asthma.

## Methods

### Patients

Patients with asthma were recruited from the Asthma Clinic of the Royal Brompton Hospital, London. Severe asthma patients underwent the Royal Brompton severe asthma protocol, in order to confirm the diagnosis and to maximise treatments[11]. All patients showed either an improvement in baseline FEV_1 _of ≥12% over baseline values after inhalation of 400 μg of salbutamol aerosol, or the presence of bronchial hyperresponsiveness defined by methacholine PC_20 _of < 4 mg/ml. Current and ex-smokers of >5 pack-years were excluded. Severe asthmatics were defined according to the American Thoracic Society major criteria of needing either continuous or near-continuous oral corticosteroids or high dose inhaled corticosteroids (2,000 μg beclomethasone-equivalent per day or more) or both in order to achieve a level of mild-moderate persistent asthma, and by 2 or more minor criteria of asthma control[12]. Patients who had well-controlled asthma defined by the lack of day-time or nocturnal symptoms and no need for reliever medications while using ≤ 800 μg of inhaled beclomethasone-equivalent per day were enrolled into the non-severe asthma group. Healthy volunteers with no diagnosis of asthma and with a negative PC_20 _(>16 mg/ml), using no medications and never-smokers, were also recruited. All participants gave informed consent to a protocol approved by the Ethics Committee of Royal Brompton & Harefield NHS Trust/National Heart & Lung Institute.

### Fiberoptic bronchoscopy

All asthmatic subjects received 5 mg of nebulised salbutamol before the procedure. Fibreoptic bronchoscopy was performed using topical anesthesia with lignocaine and intravenous sedation with midazolam. Warmed 0.9% NaCl solution (50 ml × 4) was instilled into the right middle lobe and recovery of broncho-alveolar lavage (BAL) fluid was carried out by gentle hand suction.

### Alveolar macrophage isolation

BAL cells were centrifuged (500 × g for 10 minutes) and washed with Hanks' balanced salt solution (HBSS). They were resuspended in culture media (RPMI with 0.5% fetal calf serum, antibiotics and L-glutamine) and counted using Kimura dye. Cytospins were prepared and stained with Diff Quick (Harleco, Gibbstown, NJ) stain for differential cell count. 5×10^5 ^macrophages were isolated by plastic adhesion and stimulated for 18 hours with lipopolysaccaride (LPS, 10 μg/ml) in the presence or absence of dexamethasone (Dex, 10^-6 ^M). Supernatants were aliquoted and coded. These de-identified materials were analysed in a separate laboratory for LTB_4 _and LXA_4 _by enzyme immunoassay (Cayman Chemical, Ann Arbor, Mich; Neogen, Lexington, KY). The stimulated formation of LTB_4 _and LXA_4 _was calculated as the difference between the total amount present with LPS and the basal amounts without LPS.

### Validation of Immunoreactive LXA_4 _by Addition of Authentic LXA_4_

As the absolute amounts of LXA_4 _in the macrophage supernatant samples were too low for detection by physical methods, we validated our immunoassay measurements by purposefully adding 20-30 pg of authentic LXA_4 _to selected sample aliquots and then measuring immunoreactive LXA_4 _levels in both neat and spiked samples. Addition of authentic LXA_4 _increased the total amount of LXA_4 _(endogenous plus exogenous) above the lower limits of detection for the ELISA. Neat and spiked samples displayed only minor variance in the amount of endogenous LXA_4_.

### Data analysis

Results are expressed as means ± SEM. The differences in LTB_4 _and LXA_4 _generated at baseline were compared using one-way analysis of variance with Dunn's multiple comparison test. Differences between LPS and LPS plus dexamethasone treatment were analysed using Wilcoxon paired t-tests and in this case differences between groups were compared using Mann-Whitney t-test. Correlations were performed using Spearman's rank tests. p < 0.05 was taken as significant.

## Results

Severe asthmatics had more severe airflow obstruction (p < 0.05) and bronchial hyperresponsiveness (p < 0.05) compared to non-severe asthmatics (Table 1). They were also on higher doses of inhaled corticosteroids (p < 0.05). BAL yielded fewer cells from severe asthmatics compared to non-severe asthmatics (p = 0.06), but there were proportionately more eosinophils (p < 0.05) and neutrophils (p < 0.05) with fewer macrophages (p < 0.01) in severe asthma compared to non-severe asthma.

**Table 1:** Characteristics of subjects

	Normal	Non-Severe asthma	Severe asthma
**n**	14	12	11
**Age (yr)**	21 ± 0.4	43 ± 3	47 ± 3
**Atopy**	0/14	11/12	9/11
**M/F**	12/2	7/5	4/7
**FEV_1 _**(% Predicted)	98 ± 3	85 ± 3	58 ± 6*
**PC_20 _**(mg/ml)	>16	5.73 ± 2.7	0.64 ± 0.14*
**Inhaled corticosteroid** BDP equivalent (μg/day)	0	527 ± 239 (n = 6)	2400 ± 414* (n = 11)
**Oral prednisolone (mg/day)**	0	0	19.1 ± 3.2 (n = 6)
**Total BAL Cells** (× 10^6^)	9.19 ± 1.4	8.36 ± 0.8	6.1 ± 0.99
**BAL-Macs **(×10^6^)	9.02 ± 1.32	8.17 ± 0.76	5.81 ± 1.00
**BAL-Neu **(×10^6^)	0.1 ± 0.03	0.063 ± 0.016	0.15 ± 0.03
**BAL-Eos **(×10^6^)	0.018 ± 0.012	0.026 ± 0.012	0.07 ± 0.03
**BAL-Mac (%)**	97.9 ± 0.5	97.8 ± 0.4	92.9 ± 1.5**
**BAL-Neu (%)**	1.03 ± 0.3	0.8 ± 0.2	3 ± 0.7*
**BAL-Eos (%)**	0.21 ± 0.09	0.29 ± 0.14	1.4 ± 0.55*

Abbreviations: M = Male; F = Female; BAL = Bronchoalveolar lavage; BDP = Beclomethasone dipropionate; Neu = neutrophils; Eos = eosinophils; Mac = macrophage. Data shown as mean ± SEM. *p < 0.05; **p < 0.01 compared to non-severe asthma.

### Baseline and LPS-stimulated generation of LXA_4_

**Figure 1:**
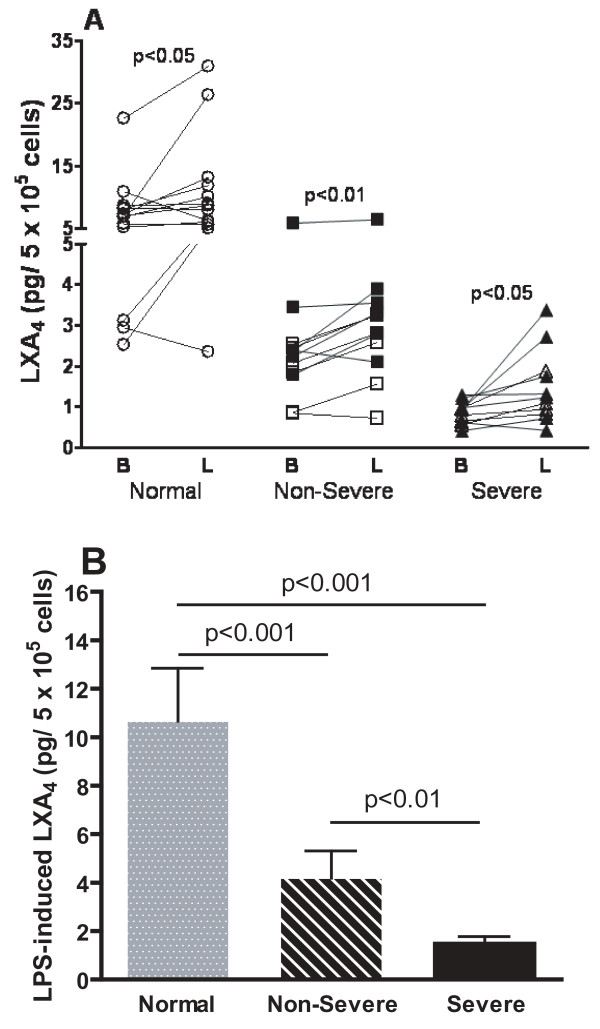
**Panel A: Individual levels of LXA_4 _in supernatants of alveolar macrophages before and after lipopolysaccharide (LPS) from normal subjects (n = 14), non-severe asthmatics (n = 12) and severe asthmatics (n = 11)**. In the asthmatic groups, closed symbols indicate those on regular treatment with inhaled and/or oral corticosteroids. Panel B: Mean levels of LXA_4 _induced by LPS (level after LPS minus basal level) in the 3 groups. Data shown as mean ± SEM.

Low levels of LXA_4 _were generated by AMs in culture. The baseline LXA_4 _from AMs obtained from normal subjects was higher than that from both non-severe (p < 0.05) and severe asthmatics (p < 0.01; Figure 1A). There was a significant difference between non-severe and severe asthmatics (p < 0.05) with a three-fold higher baseline level in non-severe asthmatics. The LXA_4 _production induced by LPS is shown as the increment in LXA_4 _above baseline (Figure 1B). There was a small but significant increase in LPS-induced LXA_4 _levels in all three groups (Figure 1B), with the lowest amounts in severe compared to non-severe asthma (p < 0.01) and to normal subjects (p < 0.001).

There was a negative correlation between baseline LXA_4 _levels in asthmatic patients and the percentage of neutrophils in the BAL (r_s _= - 0.42, p < 0.05), and a positive correlation between LPS-induced LXA_4 _levels from asthmatic patients and FEV_1 _(% predicted; r = 0.60, p < 0.01). In addition, there was a negative correlation between percentage neutrophils in the BAL and FEV_1 _(r_s _= -0.65, p < 0.001).

### Basal and LPS-stimulated generation of LTB_4_

**Figure 2:**
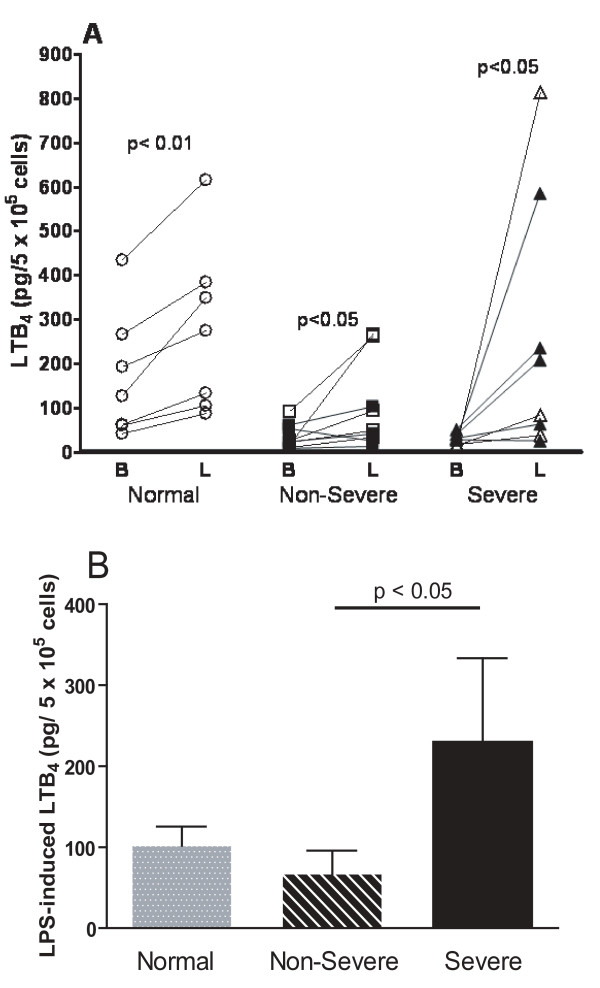
**Panel A: Individual levels of LTB_4 _in supernatants of alveolar macrophages before and after lipopolysaccharide (LPS) from normal subjects (n = 9), non-severe asthmatics (n = 9) and severe asthmatics (n = 8)**. In the asthmatic groups, closed symbols indicate those on regular treatment with inhaled and/or oral corticosteroids. Panel B: Mean levels of LPS-stimulated LTB_4 _represented by the difference between LTB_4 _levels with LPS and the basal level of LTB_4 _in the 3 groups. Data shown as mean ± SEM.

The basal level of LTB_4 _from AMs obtained from normal subjects was higher than that from both non-severe (p < 0.05) and severe asthmatics (p < 0.05; Figure 2A), with no significant differences between non-severe and severe asthmatics. The LTB_4 _production induced by LPS is shown as increments in LTB_4 _above baseline (Figure 2B). LPS induced LTB_4 _generation in all three groups (p < 0.05), but the increase in LTB_4 _in severe asthma patients was 5-fold greater than in non-severe asthmatics (p < 0.05; Figure 2B).

### Corticosteroid suppression of LXA_4_. and LTB_4_

**Figure 3:**
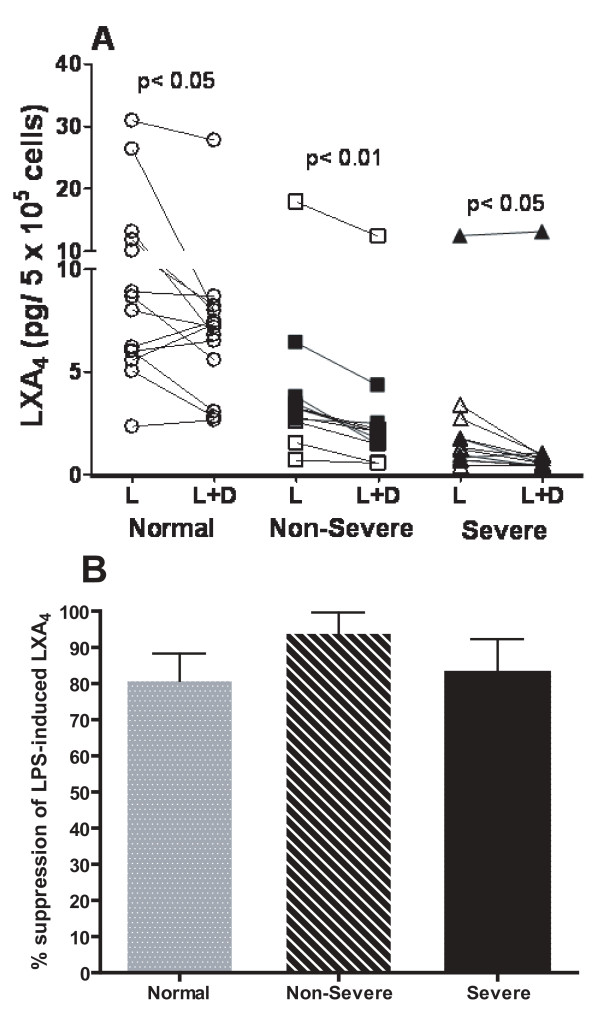
**Individual levels of LXA_4 _measured after LPS in the absence or presence of dexamethasone (10^-6 ^M) from alveolar macrophages stimulated by LPS from normal subjects (n = 14), non-severe asthmatics (n = 12) and severe asthmatics (n = 11)**. In the asthmatic groups, closed symbols indicate those on regular treatment with inhaled and/or oral corticosteroids. Data shown as mean ± SEM.

**Figure 4:**
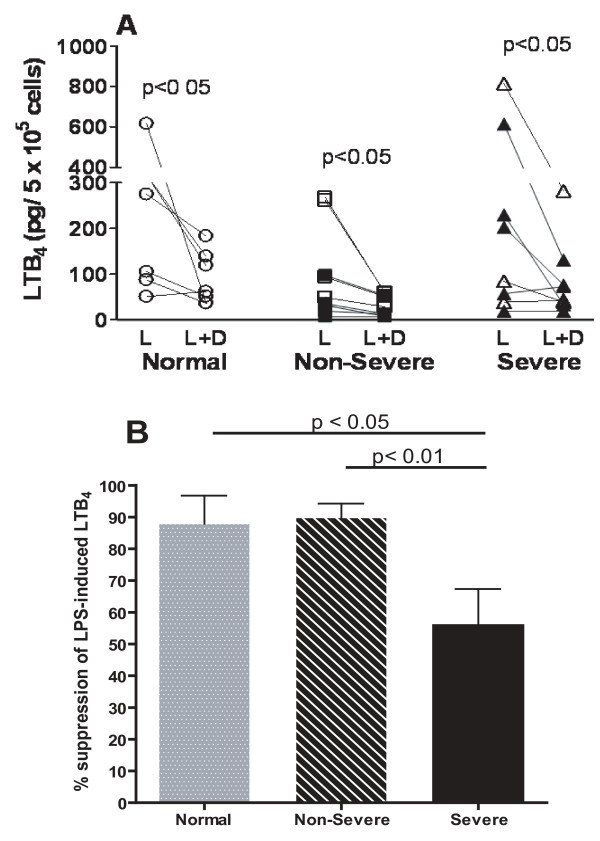
**Individual levels of LTB_4 _measured after LPS in the absence or presence of dexamethasone (10^-6 ^M) from alveolar macrophages stimulated by LPS in normal subjects (n = 9), non-severe asthmatics (n = 9) and severe asthmatics (n = 8)**. In the asthmatic groups, closed symbols indicate those on regular treatment with inhaled and/or oral corticosteroids. Data shown as mean ± SEM. Panel C. Mean degree of suppressibility of LXA_4 _and LTB_4 _release by dexamethasone. Data is expressed as % of LXA_4 _or LTB_4 _release after exposure to LPS (level after LPS minus basal level) and shown as mean ± SEM.

Dexamethasone suppression of LPS-initiated LXA_4 _was significant in all three groups (p < 0.05), with no significant differences between the groups (Figure 3). Dexamethasone suppression of LTB_4 _was observed in all three groups: normal subjects (LPS: 102 ± 23 versus LPS and dexamethasone: 11.6 ± 7.7 pg/ml, p < 0.05); non-severe asthmatics (183 ± 122 versus 21.4 ± 22 pg/ml, p < 0.05) and severe asthmatics (230 ± 102 versus 60 ± 32 pg/ml, p < 0.01). When the suppression was expressed as a percentage of LPS-induced eicosanoid production, there was no significant differences observed between normal and non-severe asthmatics with ~90% suppression. However, there was a lesser degree of suppression in severe asthmatics (Figure 4).

**Figure 5:**
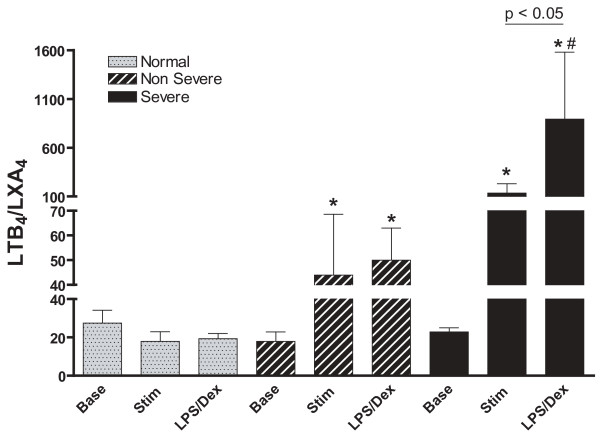
**Effect of LPS (Stim) and LPS plus dexamethasone (LPS/Dex) on the ratio of LTB_4 _to LXA_4 _from alveolar macrophages**. In both asthmatic groups, the ratio of released LTB_4 _to released LXA_4 _is increased after LPS and this is not reversed in the presence of dexamethasone. *p < 0.05 compared to baseline (base) within each group; +p < 0.01 compared to LPS/Dex of normal subjects. Data shown as shown as mean ± SEM.

In macrophages from normal subjects, the ratio of LTB_4 _to LXA_4 _(using pg/ml) was unchanged after exposure to LPS or to LPS plus dexamethasone. In non-severe asthmatics, both LPS and LPS plus dexamethasone gave an increased LTB_4_/LXA_4 _ratio, but only in severe asthmatics was the increase induced by LPS and dexamethasone significantly greater than that induced by LPS alone (p < 0.05) (Figure 5). In addition, LTB_4_/LXA_4 _ratios after LPS and dexamethasone were significantly greater in severe asthmatics compared to non-severe asthmatics (p < 0.05) as a result of both an increase in LTB_4 _and a decrease in LXA_4 _compared to normal subjects.

## Discussion

We have shown that the basal generation of the proinflammatory LTB_4 _and the anti-inflammatory LXA_4 _were both lower in cultured AMs from severe asthmatics compared to those from non-asthmatics, while only LXA_4 _was lower in severe asthmatics compared to non-severe asthmatics. The LPS induced formation of LTB_4 _was higher in severe asthma compared to non-severe asthma and normal subjects, while the LPS induced production of LXA_4 _was significantly impaired in severe asthmatics compared to normal subjects, but to a similar extent as in non-severe asthma patients. The overall effect of LPS stimulation was a net pro-inflammatory balance in terms of enhanced generation of LTB_4_, and a decrease in LXA_4 _compared to AMs from normal subjects. In addition, while the LPS-induced LTB_4 _was largely suppressed by dexamethasone, it was only partly suppressed in AMs from severe asthma patients; by contrast, induced generation of LXA_4 _was suppressed in all three groups. Therefore, the overall balance of these 2 lipid mediators in severe asthma was in favour of an overall pro-inflammatory response through both increased production and relative corticosteroid insensitivity of LTB_4 _and decreased levels of LXA_4 _in severe asthmatics, as illustrated by the LTB_4 _to LXA_4 _ratios.

Human AMs can generate both 5-lipoxygenase and 15-lipoxygenase derived eicosanoids, including LTB_4 _and LXA_4_, from endogenous sources of arachidonic acid[13]. LXA_4 _generation from endogenous stores is low, but LX biosynthesis can be amplified by select TH2 cytokines, namely interleukin-4 and interleukin-13 [14,15]. In addition, exogenous LTA_4 _can be converted by AMs to more substantial amounts of LXs [16], as would occur during transcellular biosynthesis with LTA_4 _donation from one cell to a second cell for enzymatic conversion by either 12- or 15-lipoxygenase to LXs. Our studies are the first to document both LTB_4 _and LXA_4 _generation from human AMs from asthmatic subjects. Although the levels of LXA_4 _are low, they were detected reproducibly, validated with authentic material and picogram quantities of LXs are biologically active in resolving inflammation (reviewed in reference [17]). Interestingly, when the data was expressed as a ratio of pro-inflammatory LTB_4 _to anti-inflammatory LXA_4_, there was an increased pro-inflammatory imbalance when the macrophages from both asthmatic groups were exposed to LPS, and this was not reversed by corticosteroids. Indeed, in macrophages from severe asthma, this pro-inflammatory ratio favouring LTB_4 _over LXA_4 _was further unbalanced by dexamethasone.

Our results indicate that the pulmonary macrophage can be an important source of lipid mediators, and that differences in LTB_4 _and LXA_4 _between the asthmatic groups are in general agreement with recent studies that have examined levels in whole blood[9,18] and BAL fluids [10]. In the study of *Wenzel et al*, levels of LTB_4 _in BAL fluid from severe asthma were the highest compared to levels from moderate symptomatic asthma patients and normals [19]. This indicates that the baseline contribution of LTB_4 _from macrophages is unlikely to explain this increased levels found in BAL of severe asthma patients; however, following *ex vivo *stimulation of macrophages from severe asthma patients, greater levels of LTB_4 _were released compared to non-severe asthma patients. Using a similar method as our study to distinguish severe from non-severe asthma, a deficiency in both baseline and divalent cation ionophore-stimulated production of LXA_4 _in whole blood of patients with severe asthma compared to moderate asthma was established, while the production of cysteinyl-leukotrienes and LTB_4 _were increased[9]. In addition, similar findings have been reported in airway fluids for the levels of these lipid mediators; thus, an increase in LTB_4 _levels was found in the supernatant of induced sputum of severe asthma patients compared to non-severe asthma. In these same samples, LXA_4 _levels were highest in the mild asthma group[20,21]. Moreover, LXA_4 _levels in BAL fluids from patients with severe asthma recruited in the NHLBI Severe Asthma Research Program are decreased compared to non-severe asthma patients, and BAL cells from severe asthma patients had increased 5-LO and decreased 15-LO expression [10]. These results are in line with the current observation of reduced basal and LPS-stimulated production of LXA_4 _from alveolar macrophages of patients with severe asthma. In conjunction with the large number of alveolar macrophages in healthy and asthmatic lung, these observations provide support to the idea that the alveolar macrophage is a likely important source of LXA_4 _in human airways.

There have been very few studies of the effect of LPS on human macrophages in terms of LT and LX generation. Brief exposure of murine macrophages to LPS can prime them to increase LT synthesis in response to an activating stimulus such as immune complexes or divalent cation ionophore A23187[22], an observation that has been subsequently shown in human AMs [23]. On the other hand, prolonged exposure to LPS impaired the capacity of rat macrophages to produce LTs in response to stimulating agents, a process that was due to the production of inhibitory substances such as nitric oxide [24,25]. LPS can induce human AM phagocytosis of apoptotic cells, but AMs from subjects with severe asthma display defective clearance mechanisms and lower levels of PGE_2 _and 15-hydroxyeicosatetraenoic acid formation in response to LPS[26]. Both PGE_2 _and 15-HETE can play pivotal roles in establishing LX biosynthesis [27,28].

Differences in basal LTB_4 _from AMs have not been previously observed between asthma and normal atopic or non-atopic control subjects[29], or those with nocturnal asthma [30]. However, asthmatic subjects in these studies would not have met the NHLBI Severe Asthma Research Program's criteria for severe asthma [2]. Regarding calcium ionophore-induced LTB_4 _biosynthesis by AMs, one study reported increased LTB_4 _generation by cells in asthma compared to non-asthmatics [31], while another study did not report any significant differences[29]. Our study shows reduced baseline LTB_4 _in non-severe asthma and no significant differences in stimulated production by LPS compared to non-asthmatics. Because LTB_4 _biosynthesis by AMs *in vitro *can be modulated by environmental factors *in vivo*, such as cigarette smoking [32], smokers were excluded from the study.

Glucocorticoids have been shown to inhibit LT generation through inhibition of phospholipase A_2 _activity [33,34]. Chronic oral corticosteroid therapy may lead to a suppression of eicosanoid biosynthesis and could underlie the baseline reduction in LXA_4 _and LTB_4 _observed in the macrophages from patients with severe asthma. Both LTB_4 _and LXA_4 _stimulated by calcium ionophore in the circulating neutrophil was reduced in corticosteroid-dependent asthmatics who were on oral prednisolone [35]. However, as far as the AM is concerned, there was no significant inhibition of LTB_4 _from AMs of normal subjects treated with oral prednisolone despite the fact that direct incubation of these cells with dexamethasone leads to an inhibition of basal and calcium ionophore triggered formation of LTB_4 _[36]. Other work also indicate that oral short-term treatment with prednisone does not inhibit the levels of the eicosanoids, PGD_2_, 5-HETE and LTE_4_, in BAL from asthmatic subjects at baseline or after allergen challenge [37]. However, *ex-vivo *treatment of BAL cells with prednislone did cause inhibition of LTB_4 _and thromboxane generation. Similarly, in the work of Wenzel *et al*, a single dose of oral prednisone inhibited LTB_4 _release from alveolar macrophages from patients with nocturnal asthma but not from those without nocturnal asthma [30]. Only half of the patients with severe asthma in this study were on oral corticosteroid therapy and there was no significant differences in terms of LPS-induced LTB_4 _or LXA_4 _or of dexamethasone-induced suppression between macrophages of severe asthma patients who were on prednisolone versus those not on prednisolone. Similarly, in the non-severe asthma group, there was no difference in terms of LPS-induced LTB_4 _or LXA_4 _or of dexamethasone-induced suppression between macrophages of non-severe asthma patients who were on daily inhaled corticosteroids versus those not on inhaled corticosteroids. However, the influence of long-term oral or inhaled corticosteroid therapy, as contrasted to short-term, on this *ex-vivo *production of arachidonic acid-derived mediators cannot be entirely excluded.

We have elected to group our asthmatic patients as non-severe and severe asthma patients on the basis of the definition of severe asthma proposed by the ATS [12]. This definition of severe asthma is based on the lack of control of asthma despite taking maximal anti-inflammatory treatments with corticosteroids, while the non-severe asthma patients were those on no or only low-dose inhaled corticosteroids. We observed that there was relative CS insensitivity of LTB_4 _generation but not of LXA_4 _from AMs of patients with severe asthma. Previously, no differences in CS sensitivity of AMs in terms of calcium ionophore induced LTB_4 _from asthmatics as compared to non-asthmatic macrophages have been reported[29]. In a previous study, we have shown that AMs from patients with severe asthma demonstrate a reduced sensitivity to dexamethasone in terms of LPS-induced release of pro-inflammatory cytokines [5].

Our data on LXA_4 _is one of the first regarding its stimulated production by LPS, and its suppression by dexamethasone. Levels of anti-inflammatory LXs were low in severe asthmatics, and did not increase in response to LPS stimulation, further increasing the disparity between severe asthmatics and non-severe asthmatics in the levels of these mediators. Moreover, dexamethasone suppressed LPS-induced increases in LXA_4 _in all groups. This differential response of AMs from severe asthmatics vis-a-vis LTB_4 _and LXA_4 _and the effect of corticosteroids (increased LTB_4 _in response to LPS and impaired CS suppression of the rise in LTB_4 _vs. little change in LXA_4 _in response to LPS and unimpaired CS suppression of LXA_4 _levels) may contribute to persistent airway neutrophilic inflammation since LXA_4 _can inhibit LTB_4_-induced chemotaxis, adhesion and transmigration[17]. This potential role of LXA_4 _in regulating neutrophil chemotaxis is supported by the inverse relationship between baseline LXA_4 _and the percentage of neutrophils in bronchoalveolar lavage fluid.

Lipoxins are a distinct class of eicosanoids with anti-inflammatory properties at subnanomolar concentrations. Thus, although the basal and stimulated levels of LXA_4 _from alveolar macrophages are in low picogram amounts, these levels would be predicted to have pro-resolving actions for airway inflammation (reviewed in [17]. In support of the protective effect of LXA_4_, we found a positive correlation between LPS-induced LXA_4 _and lung function as represented by FEV_1_. Indirectly, this protection in lung function may occur through an effect on neutrophilic inflammation, since there was an inverse correlation between BAL neutrophilia and FEV_1_. LXA_4 _can inhibit LTB_4_-initiated chemotaxis, adhesion and transmigration. In addition, LXA_4 _inhibits eosinophilic allergic inflammation [38]. Thus, a possible imbalance in LTB_4 _and LXA_4 _in the airways would serve to increase airway neutrophil and eosinophil accumulation and activation. Interestingly, a similar imbalance between LT and LX generation is present in scleroderma lung disease [39] and decreased lipoxin production has also been reported in inflammatory bowel disease [40].

One of the potential limitations of our work regards the relative age of the healthy control group that were younger than the asthma groups. Generation of LXA_4 _can decrease and LTB_4 _increase with age [41,42], but there is no information available at present on the influence of age on the release of these eicosanoids from human alveolar macrophages. While there may be uncertainty about the effect of age, we are able to compare the non-severe with the severe group of asthmatic subjects as they were of comparable age group.

In summary, we demonstrate impaired corticosteroid modulation of the pro-inflammatory lipid mediator LTB_4 _but not of the anti-inflammatory lipoxin, LXA_4_, in AMs of severe asthma. Together with the augmented LPS induced formation of LTB_4 _and decreased generation of LXA_4 _in severe asthma, our observations indicate a net pro-inflammatory imbalance in severe asthma.

## Abbreviations

AM: alveolar macrophage; BAL: bronchoalveolar lavage; BALF: bronchoalveolar lavage fluid; CS: corticosteroid; Dex: dexamethasone; FEV_1_: forced expiratory volume in one second; LT: leukotriene; LTB_4_: leukotriene B_4 _LPS: lipopolysaccaride; LX: liopoxin; LXA_4_: lipoxin A_4_; PC_20_: provocative concentration of metacholine causing a 20% fall in FEV_1_; SEM: standard error of the mean.

## Competing interests

PB has no conflicts of interest to disclose. BBL is a co-inventor on patents on lipoxins that are owned by Brigham and Women's Hospital that have been licensed for clinical development and are the subject of consultancies. MH has no conflicts of interest to disclose. MP has no conflicts of interest to disclose. SK has no conflicts of interest to disclose. EI has no conflicts of interest to disclose. KFC has participated on Advisory Boards of several pharmaceutical companies to discuss treatments used for asthma and COPD. He has received unrestricted grant money from one pharmaceutical company, and other grant money to participate in clinical trials.

## Authors' contributions

KFC and BDL conceived the study, PB and MH collected the samples, MP, SK and BDL did the measurements of lipoxins, PB, BDL, EI and KFC wrote the manuscript. All the authors have read the and approved the final manuscript.

## References

[B1] Stirling RG Chung KF Severeasthma: definition and mechanisms Allergy 2001 56 825 840 10.1034/j.1398-9995.2001.00143.x 11551247

[B2] Moore WC Bleecker ER Curran-Everett D Erzurum SC Ameredes BT Bacharier L Calhoun WJ Castro M Chung KF Clark MP Dweik RA Fitzpatrick AM Gaston B Hew M Hussain I Jarjour NN Israel E Levy BD Murphy JR Peters SP Teague WG Meyers DA Busse WW Wenzel SE Characterization of the severe asthma phenotype by the National Heart, Lung, and Blood Institute's Severe Asthma Research Program J Allergy Clin Immunol 2007 119 405 413 10.1016/j.jaci.2006.11.639 17291857 PMC2837934

[B3] Jatakanon A Uasuf C Maziak W Lim S Chung KF Barnes PJ Neutrophilic inflammation in severe persistent asthma Am J Respir Crit Care Med 1999 160 1532 1539 10556116 10.1164/ajrccm.160.5.9806170

[B4] Wenzel SE Schwartz LB Langmack EL Halliday JL Trudeau JB Gibbs RL Chu HW Evidence That Severe Asthma Can Be Divided Pathologically into Two Inflammatory Subtypes with Distinct Physiologic and Clinical Characteristics Am J Respir Crit Care Med 1999 160 1001 1008 10471631 10.1164/ajrccm.160.3.9812110

[B5] Bhavsar P Hew M Khorasani N Torrego A Barnes PJ Adcock I Chung KF Relative corticosteroid insensitivity of alveolar macrophages in severe asthma compared with non-severe asthma Thorax 2008 63 784 790 10.1136/thx.2007.090027 18492738

[B6] Hew M Bhavsar P Torrego A Meah S Khorasani N Barnes PJ Adcock I Fan Chung K National Heart Lung and Blood Institute's Severe Asthma Research Program Relative Corticosteroid Insensitivity of Peripheral Blood Mononuclear Cells in Severe Asthma Am J Respir Crit Care Med 2006 174 134 141 10.1164/rccm.200512-1930OC 16614347 PMC2662905

[B7] Chung KFBP Cytokines in asthma Thorax 1999 54 825 857 10.1136/thx.54.9.825 10456976 PMC1745579

[B8] Levy BD De Sanctis GT Devchand PR Kim E Ackerman K Schmidt BA Szczeklik W Drazen JM Serhan CN Multi-pronged inhibition of airway hyper-responsiveness and inflammation by lipoxin A(4) Nat Med 2002 8 1018 1023 10.1038/nm748 12172542

[B9] Levy BD Bonnans C Silverman ES Palmer LJ Marigowda G Israel E Severe Asthma Research Program NHLaBI Diminished Lipoxin Biosynthesis in Severe Asthma Am J Respir Crit Care Med 2005 172 824 830 10.1164/rccm.200410-1413OC 15961693 PMC2718403

[B10] Planaguma A Kazani S Marigowda G Haworth O Mariani TJ Israel E Bleecker ER Curran-Everett D Erzurum SC Calhoun WJ Castro M Chung KF Gaston B Jarjour NN BUSSE WW Wenzel SE Levy BD NHLBI Severe Asthma Research Program (SARP) Airway Lipoxin A4 Generation and Lipoxin A4 Receptor Expression Are Decreased in Severe Asthma Am J Respir Crit Care Med 2008 178 574 582 10.1164/rccm.200801-061OC 18583575 PMC2542432

[B11] Robinson DS Campbell DA Durham SR Pfeffer J Barnes PJ Chung KF Systematic assessment of difficult-to-treat asthma Eur Respir J 2003 22 478 483 10.1183/09031936.03.00017003 14516138

[B12] Proceedings of the ATS Workshop on Refractory Asthma. Current Understanding, Recommendations, and Unanswered Questions Am J Respir Crit Care Med 2000 162 2341 2351 11112161 10.1164/ajrccm.162.6.ats9-00

[B13] Levy BD Romano M Chapman HA Reilly JJ Drazen J Serhan CN Human alveolar macrophages have 15-lipoxygenase and generate 15(S)-hydroxy-5,8,11-cis-13-trans-eicosatetraenoic acid and lipoxins J Clin Invest 1993 92 1572 1579 10.1172/JCI116738 8376607 PMC288306

[B14] Nassar GM Morrow JD Roberts LJ Lakkis FG Badr KF Induction of 15-lipoxygenase by interleukin-13 in human blood monocytes J Biol Chem 1994 269 27631 27634 7961680

[B15] Profita M Sala A Siena L Henson PM Murphy RC Paterno A Bonanno A Riccobono L Mirabella A Bonsignore G Vignola AM Leukotriene B4 Production in Human Mononuclear Phagocytes Is Modulated by Interleukin-4-Induced 15-Lipoxygenase J Pharmacol Exp Ther 2002 300 868 875 10.1124/jpet.300.3.868 11861792

[B16] Chavis C Godard P de Crastes Paulet A Damon M Formation of lipoxins and leukotrienes by human alveolar macrophages incubated with 15(S)-HETE: a model for cellular cooperation between macrophages and airway epithelial cells Eicosanoids 1992 5 203 211 1337977

[B17] Serhan CN Resolution Phase of Inflammation: Novel Endogenous Anti-Inflammatory and Proresolving Lipid Mediators and Pathways Annual Review of Immunology 2007 25 101 137 10.1146/annurev.immunol.25.022106.141647 17090225

[B18] Çelik GE Erkekol FO Misirligil Z Melli M Lipoxin A_4 _levels in asthma: relation with disease severity and aspirin sensitivity Clin Exp Allergy 2007 37 1494 1501 17883729 10.1111/j.1365-2222.2007.02806.x

[B19] Wenzel S Szefler S Leung D Sloan S Rex M Martin R Bronchoscopic Evaluation of Severe Asthma. Persistent Inflammation Associated with High Dose Glucocorticoids Am J Respir Crit Care Med 1997 156 737 743 9309987 10.1164/ajrccm.156.3.9610046

[B20] Vachier I Bonnans C Chavis C Farce M Godard P Bousquet J Chanez P Severe asthma is associated with a loss of LX4, an endogenous anti-inflammatory compound J Allergy Clin Immunol 2005 115 55 60 10.1016/j.jaci.2004.09.038 15637547

[B21] Bonnans C Vachier I Chavis C Godard P Bousquet J Chanez P Lipoxins Are Potential Endogenous Antiinflammatory Mediators in Asthma Am J Respir Crit Care Med 2002 165 1531 1535 10.1164/rccm.200201-053OC 12045128

[B22] Aderem AACDWSCZ Bacterial lipopolysaccharides prime macrophages for enhanced release of arachidonic acid metabolites J Exp Med 1986 164 179 10.1084/jem.164.1.165 PMC2188197 2941513

[B23] Suzuki K Yamamoto T Sato A Murayama T Amitani R Yamamoto K Kuze F Lipopolysaccharide primes human alveolar macrophages for enhanced release of superoxide anion and leukotriene B4: self-limitations of the priming response with protein synthesis Am J Respir Cell Mol Biol 1993 8 500 508 8386927 10.1165/ajrcmb/8.5.500

[B24] Coffey MJ Phare SM Peters-Golden M Prolonged Exposure to Lipopolysaccharide Inhibits Macrophage 5-Lipoxygenase Metabolism Via Induction of Nitric Oxide Synthesis J Immunol 2000 165 3592 3598 11034360 10.4049/jimmunol.165.7.3592

[B25] Brock TG McNish RW Mancuso P Coffey MJ Peters-Golden M Prolonged lipopolysaccharide inhibits leukotriene synthesis in peritoneal macrophages: mediation by nitric oxide and prostaglandins Prostaglandins Other Lipid Mediat 2003 71 131 145 10.1016/S1098-8823(03)00036-4 14518557

[B26] Huynh ML Malcolm KC Kotaru C Tilstra JA Westcott JY Fadok VA Wenzel SE Defective Apoptotic Cell Phagocytosis Attenuates Prostaglandin E2 and 15-Hydroxyeicosatetraenoic Acid in Severe Asthma Alveolar Macrophages Am J Respir Crit Care Med 2005 172 972 979 10.1164/rccm.200501-035OC 16040786

[B27] Levy BD Clish CB Schmidt B Gronert K Serhan CN Lipid mediator class switching during acute inflammation: signals in resolution Nat Immunol 2001 2 612 619 10.1038/89759 11429545

[B28] Brezinski ME Serhan CN Selective incorporation of (15S)-hydroxyeicosatetraenoic acid in phosphatidylinositol of human neutrophils: agonist-induced deacylation and transformation of stored hydroxyeicosanoids Proc Natl Acad Sci USA 1990 87 6248 6252 10.1073/pnas.87.16.6248 2117277 PMC54510

[B29] Balter MS Eschenbacher WL Peters-Golden M Arachidonic acid metabolism in cultured alveolar macrophages from normal, atopic, and asthmatic subjects Am Rev Respir Dis 1988 138 1134 1142 3144211 10.1164/ajrccm/138.5.1134

[B30] Wenzel SE Trudeau JB Westcott JY Beam WR Martin RJ Single oral dose of prednisone decreases leukotriene B4 production by alveolar macrophages from patients with nocturnal asthma but not control subjects: relationship to changes in cellular influx and FEV1 J Allergy Clin Immunol 1994 94 870 881 10.1016/0091-6749(94)90155-4 7963156

[B31] Damon M Chavis C Daures JP Crastes dP Michel FB Godard P Increased generation of the arachidonic metabolites LTB4 and 5-HETE by human alveolar macrophages in patients with asthma: effect in vitro of nedocromil sodium Eur Respir J 1989 2 202 209 2543595

[B32] Laviolette M Coulombe R Picard S Braquet P Borgeat P Decreased leukotriene B4 synthesis in smokers' alveolar macrophages in vitro J Clin Invest 1986 77 54 60 10.1172/JCI112301 3003154 PMC423308

[B33] Sorensen D Kelly T Murray D Nelson D Corticosteroids stimulate an increase in phospholipase A2 inhibitor in human serum J Steroid Biochem 1988 29 271 273 10.1016/0022-4731(88)90276-2 3347066

[B34] Sano A Munoz N Sano H Choi J Zhu X Jacobs B Leff A Inhibition of cPLA2 Translocation and Leukotriene C4 Secretion by Fluticasone Propionate in Exogenously Activated Human Eosinophils Am J Respir Crit Care Med 1999 159 1903 1909 10351938 10.1164/ajrccm.159.6.9810005

[B35] Vachier I Chavis C Majori M Farce M Bousquet J Godard P Chanez P Effects of glucocorticoids on endogenous and transcellular metabolism of eicosanoids in asthma Journal of Allergy and Clinical Immunology 2001 107 824 831 10.1067/mai.2001.113868 11344349

[B36] Yoss EB Spannhake EW Flynn JT Fish JE Peters SP Arachidonic acid metabolism in normal human alveolar macrophages: stimulus specificity for mediator release and phospholipid metabolism, and pharmacologic modulation in vitro and in vivo Am J Respir Cell Mol Biol 1990 2 69 80 2155013 10.1165/ajrcmb/2.1.69

[B37] Dworski R Fitzgerald GA Oates JA Sheller JR Effect of oral prednisone on airway inflammatory mediators in atopic asthma Am J Respir Crit Care Med 1994 149 953 959 8143061 10.1164/ajrccm.149.4.8143061

[B38] Levy BD Lukacs NW Berlin AA Schmidt B Guilford WJ Serhan CN Parkinson JF Lipoxin A4 stable analogs reduce allergic airway responses via mechanisms distinct from CysLT1 receptor antagonism FASEB J 2007 21 3877 3884 10.1096/fj.07-8653com 17625069 PMC3005621

[B39] Kowal-Bielecka O Kowal K Distler O Rojewska J Bodzenta-Lukaszyk A Michel BA Gay RE Gay S Sierakowski S Cyclooxygenase-lipoxygenase-derived eicosanoids in bronchoalveolar lavage fluid from patients with scleroderma lung disease: An imbalance between proinflammatory and antiinflammatory lipid mediators Arthritis and rheumatism 2005 52 3783 3791 10.1002/art.21432 16320329

[B40] Mangino MJ Brounts L Harms B Heise C Lipoxin biosynthesis in inflammatory bowel disease Prostaglandins & Other Lipid Mediators 2006 79 84 92 10.1016/j.prostaglandins.2005.10.004 16516812

[B41] Lu MC Peters-Golden M Hostetler DE Robinson NE Derksen FJ Age-related enhancement of 5-lipoxygenase metabolic capacity in cattle alveolar macrophages Am J Physiol Lung Cell Mol Physiol 1996 271 L547 L554 10.1152/ajplung.1996.271.4.L547 8897901

[B42] Gangemi S Pescara L D'Urbano E Basile G Nicita-Mauro V Dav¥ G Romano M Aging is characterized by a profound reduction in anti-inflammatory lipoxin A4 levels Experimental Gerontology 2005 40 612 614 10.1016/j.exger.2005.04.004 15935589

